# Predictors of Mortality in Burn Patients at Selected Tertiary Public Hospitals in Addis Ababa, Ethiopia: A Two-Year Retrospective Study

**DOI:** 10.3390/ebj7020028

**Published:** 2026-05-12

**Authors:** Rahel Kassa Bayou, Meheret Befekadu Demmissie, Bethelhem Kassa Bayou, Laura Pompermaier, Hanna Yemane Berhane, Bacha Mirkena Dhabi

**Affiliations:** 1 Plastic and Reconstructive Surgery Unit, Department of Surgery, Faculty of Medicine, Saint Paul’s Hospital Millennium Medical College (SPHMMC), Addis Ababa P.O. Box 1271, Ethiopia; 2 ALERT Comprehensive Specialized Hospital (All Africa Leprosy, Tuberculosis, and Rehabilitation Training Center), Addis Ababa P.O. Box 165, Ethiopia; meheretbd@gmail.com (M.B.D.); bethyekbtt@gmail.com (B.K.B.); 3 Centre Teaching and Research in Disaster Medicine and Traumatology (KMC), Department of Biomedical and Clinical Sciences, Linköping University, 581 83 Linköping, Sweden; laura.pompermaier@liu.se; 4 Department of Biomedical and Clinical Sciences, Division of Surgery, Orthopedics and Oncology, Linköping University, 581 85 Linköping, Sweden; 5 Department of Hand and Plastic Surgery and Burns, University Hospital, 581 85 Linköping, Sweden; 6 Nutrition and Behavioral Sciences Department, Addis Continental Institute of Public Health, Addis Ababa P.O. Box 26751/1000, Ethiopia; hannayaciph@gmail.com; 7 Department of Outpatient, Addis Ababa Burn, Emergency and Trauma (AaBET) Hospital, Affiliate of Saint Paul’s Hospital Millennium Medical College (SPHMMC), Addis Ababa P.O. Box 1271, Ethiopia; bacmirle2004@gmail.com

**Keywords:** burn injury, mortality, inhalation injury, predictors, retrospective study, Ethiopia

## Abstract

**Highlights:**

**What are the main findings?**

- Burn injuries disproportionately affect children and young adults, with scalds as the leading cause.
- In-hospital mortality was 8.5%, strongly predicted by inhalation injury, TBSA ≥ 15%, and ICU admission.

**What are the implications of the main findings?**

- Strengthening critical care and burn management capacity is urgently needed.
- Prevention and treatment strategies should prioritize children and young adults at highest risk.

**Abstract:**

**Background:** Burn injuries are a major cause of morbidity and mortality in low- and middle-income countries, yet remain underreported due to limited data systems. This study describes the epidemiology of burn patients admitted to two major burn centers in Addis Ababa, Ethiopia, and identifies predictors of in-hospital mortality. **Methods:** A cross-sectional study was conducted among patients with new burn injuries admitted between 1 September 2021, and 1 November 2023, at the Addis Ababa Burn, Emergency, and Trauma Center (AaBET) and Yekatit 12 Medical College (Y12MC) hospitals. Data were extracted from medical records. Descriptive statistics summarized patient characteristics, and binary logistic regression with multivariable analysis identified factors associated with in-hospital mortality using adjusted odds ratios (AORs) and 95% confidence intervals (CIs). **Results:** Chart completeness was 96.2%. Among 800 patients, 57% were female, with a median age of 18 years (range: 0–89); approximately 80% were under 30 years. Scalds were the leading cause (49.1%). In-hospital mortality was 8.5% (95% CI: 6.5–10.4). Significant predictors included inhalation injury (AOR 6.53), TBSA ≥ 15% (AOR 3.33), deep burns (AOR 1.96), and ICU admission (AOR 14.42). **Conclusions:** In-hospital mortality was moderate, disproportionately affecting children and young adults, underscoring the need to strengthen critical care and management of severe burns.

## 1. Introduction

Burn injury is a type of tissue damage caused by exposure to thermal, electrical, chemical, or radiation energy and remains a major cause of preventable injury worldwide [1]. Burns represent a significant global public health challenge, particularly in low- and middle-income countries (LMICs), where access to timely and specialized burn care is often limited [2]. According to the World Health Organization, an estimated 180,000 deaths occur annually due to burn injuries, with the vast majority occurring in LMICs [1]. Beyond mortality, burns are associated with prolonged hospitalization, permanent disability, psychological trauma, and substantial socioeconomic consequences, disproportionately affecting children and economically productive age groups [3,4,5].

In Africa, burn injuries account for a substantial proportion of trauma-related morbidity and mortality. Studies from sub-Saharan Africa demonstrate a persistently high burden of severe burns, with hospital-based case fatality rates averaging around 17%, substantially higher than in high-income countries [6,7]. This elevated mortality is influenced not only by burn severity but also by broader health system challenges, including delayed presentation, limited intensive care capacity, inadequate burn units, and shortages of trained personnel and essential supplies [6,7,8].

In Ethiopia, burn injuries are increasingly recognized as a major public health problem and a significant contributor to trauma-related mortality [7]. Burn care is delivered through a limited number of specialized centers, including Addis Ababa Burn, Emergency and Trauma (AaBET) Hospital and Yekatit 12 Medical College (Y12MC), which serve as major referral institutions for moderate to severe burn injuries [9]. Despite their critical role, contemporary facility-based data on burn outcomes and associated factors remain limited and heterogeneous across settings [10,11].

Therefore, this study aimed to determine in-hospital mortality among burn patients admitted to these centers and to identify clinical and demographic factors associated with mortality.

## 2. Materials and Methods

A facility-based cross-sectional study was conducted at two public referral centers providing specialized burn care in Addis Ababa, Ethiopia: AaBET and Y12MC Hospitals.

Y12MC Hospital, operating under the Addis Ababa City Administration Health Bureau, is a long-established burn care center with a dedicated 19-bed burn unit (12 adult and 7 pediatric beds). AaBET Hospital, affiliated with St. Paul’s Hospital Millennium Medical College (SPHMMC), was established in 2016 to address gaps in emergency and surgical care. Its burn unit, under the Department of Plastic and Reconstructive Surgery, also has a 19-bed capacity (12 adult and 7 pediatric beds).

Although no formal national burn referral guidelines exist, patients are typically referred to these centers for moderate to severe burns, inhalation injuries, burns involving critical areas (face, joints), chemical or electrical burns, and burn-related complications such as infection or sepsis. Y12MC Hospital primarily serves patients from Addis Ababa and nearby regions, while AaBET functions as a national referral center receiving severe burn cases from across the country.

### 2.1. Health System Structure and Referral Pathways

Ethiopia’s healthcare system is organized into a three-tier structure comprising primary, secondary, and tertiary levels of care [12,13,14]. At the primary level, Primary Health Care Units (PHCUs) include health posts, health centers, and primary hospitals [12,13]. Health posts serve as the first point of contact at the community level and primarily provide preventive and basic curative services, while health centers provide outpatient care, emergency services, and initial stabilization prior to referral to higher-level facilities [12,13,14]. Health centers represent the next level of care and function as the first point of formal clinical management for most patients [12]. These facilities provide outpatient services, emergency care, and initial stabilization, including basic burn management such as fluid resuscitation, wound care, analgesia, and referral coordination prior to transfer to higher-level hospitals [12,13,14]. In this study, the first point of care was categorized as health center (public PHCU level); private clinic or primary hospital; other tertiary hospital (excluding study sites); and self-referral.

### 2.2. Study Population

The study included all patients of any age and sex with acute burn injuries admitted to the selected hospitals between 1 September 2021, and 1 November 2023. Exclusion criteria were: Missing or irretrievable medical charts, incomplete documentation of key study variables, Patients documented as leaving against medical advice.

### 2.3. Patient Management Protocol

At both institutions, burn patients are initially assessed and resuscitated in the Emergency Department by emergency physicians. Fluid resuscitation for major burns is guided by the Parkland formula, a well-established protocol. Patients requiring ongoing care are admitted to the burn unit following initial stabilization. Patients enrolled in Community-Based Health Insurance (CBHI) receive inpatient burn care free of charge, whereas uninsured patients cover treatment costs out-of-pocket.

### 2.4. Sample Size

The required sample size was initially calculated using a single population proportion formula, yielding 345 patients. However, to enhance representativeness and statistical power, all eligible patient charts within the study period were included. Consequently, a total of 832 patient records were analyzed.

### 2.5. Data Sources and Extraction

Data were extracted from secondary sources, specifically the Emergency Health Management Information System (HMIS) logbooks and patient medical records.

The data extraction tool included: Sociodemographic variables: age, sex, residence (urban/rural); Clinical variables: time to presentation, cause of burn, total body surface area burned (TBSA%), burn depth, anatomical site, inhalation injury, comorbidities; Treatment and outcome variables: length of hospital stay, ICU admission, and in-hospital mortality.

### 2.6. Operational Definitions

The following operational definitions were applied in this study:

○ Rural within 2 h: Patients residing in rural areas located within approximately a two-hour travel time to Addis Ababa.
○ Rural >2 h: Patients residing in rural areas requiring more than two hours of travel time to reach Addis Ababa.
○ Time to care <8 h: Patients who sought initial medical care within eight hours of sustaining the burn injury.

### 2.7. Data Collection and Quality Assurance

Data were collected using a structured, paper-based checklist by two trained nurses. Training was provided on data collection procedures, ethical considerations, and tool utilization.

Data quality was ensured through daily supervision by the principal investigator, including checks for completeness, consistency, and accuracy. Data was entered into Epi Info version 7.2.6.0 and exported to SPSS version 27 for cleaning and analysis.

### 2.8. Statistical Analysis

Descriptive statistics (frequency, percentage, median, and range) were used to summarize patient characteristics. Binary logistic regression analysis was performed to identify predictors of in-hospital mortality. Variables with a *p*-value < 0.2 in bivariate analysis were included in the multivariable model. Statistical significance was set at *p* < 0.05, and associations were reported using adjusted odds ratios (AORs) with 95% confidence intervals.

### 2.9. Data Availability

The dataset used and/or analyzed during the current study is available from the corresponding author upon reasonable request. There are no restrictions on data accessibility; however, patient confidentiality is strictly maintained through de-identification of all records.

### 2.10. Ethical Considerations

Ethical approval was obtained from the Institutional Review Board of Addis Continental Institute of Public Health (protocol number: AC_IPHRO/0014/2024), the Research Directorate of SPHMMC, and the Addis Ababa Public Health Research and Emergency Management Directorate (reference number: A/A/9881/227). As the study utilized retrospective patient records, informed consent was waived. All data were anonymized to ensure confidentiality. No generative artificial intelligence tools were used in study design, data collection, analysis, or interpretation. AI-assisted tools were used only for language editing and formatting, which does not require formal disclosure under journal guidelines.

## 3. Results

Following the exclusion of 32 patients due to incomplete data, a total of 800 patients with new burn injuries were included in the analysis. These patients were admitted to two burn centers in Addis Ababa during the study period.

### 3.1. Sociodemographic Characteristics

Of the total study population, 459 patients (57.4%) were admitted to Y12MC Hospital, while 341 (42.6%) were admitted to AaBET Hospital. The majority of patients (86.8%) were from urban areas. The median age was 18 years (range: 9 months–90 years), with children under 10 years representing the largest proportion (36.0%). Females accounted for 57.0% of the cohort, resulting in a male-to-female ratio of 1:1.3 (See Table 1).

### 3.2. Clinical Findings and Burn Characteristics

The median Total Body Surface Area (TBSA) burned was 15% (IQR: 8–22). Inhalation injury was identified in 112 patients (14.0%). Scald burns were the most common mechanism (49.1%), followed by flame burns (24.5%) and electrical burns (24.4%). Most patients (80.6%) presented within 8 h of injury, and 57.5% initially received care at other health centers before referral. Pre-existing comorbidities were reported in 56 patients, with epilepsy and hypertension being the most common (each 3.0%). The most frequently affected anatomical sites among burn patients were the upper extremities (28.1%), followed by the head and neck combined (16.9%), the head alone (15.3%), and the trunk (12.6%). Other affected regions included the lower extremities (7.8%), the perineum (2.1%), and multiple body regions (13.0%), while isolated neck burns (4.3%) were the least common (See Table 2).

### 3.3. Patient Outcomes

A total of 45 patients (5.6%) required admission to the Intensive Care Unit (ICU). The overall in-hospital mortality rate was 8.5% (95% CI: 6.5–10.4), corresponding to 68 deaths. Of these deaths, 39 occurred at Y12MC Hospital and 29 at AaBET Hospital. The majority of deaths occurred among female patients (*n* = 42). The leading causes of death documented in the medical records were septic shock *(n* = 30), followed by hospital-acquired or ventilator-associated pneumonia (*n* = 23), and multi-organ failure (*n* = 15).

### 3.4. Factors Associated with Burn-Related Mortality

#### 3.4.1. Bivariable Analysis

In the bivariable logistic regression analysis, several variables met the inclusion criteria (*p* < 0.2) for inclusion in the multivariable model. These factors included burn depth, inhalation injury, mechanism of burn, total body surface area (TBSA) burned, place of hospital stay, head and neck involvement, and source of referral.

#### 3.4.2. Multivariable Logistic Regression Analysis

In the multivariable model, five variables demonstrated a statistically significant association with burn-related mortality. Patients with deep burns (AOR = 1.97; 95% CI: 1.01–3.84), inhalation injury (AOR = 6.53; 95% CI: 2.14–19.95), TBSA ≥ 15% (AOR = 3.33; 95% CI: 1.29–8.61), and ICU admission (AOR = 14.42; 95% CI: 5.87–35.40) had significantly higher odds of death. Conversely, referral from another health facility (AOR = 0.18; 95% CI: 0.053–0.61) was associated with reduced odds of mortality (See Table 3).

## 4. Discussion

This study analyzed burn mortality and associated factors at two major burn referral hospitals in Addis Ababa over a two-year period. The overall in-hospital mortality rate was 8.5%, which is comparable to reports from Tanzania (7.1%) [15] and Ethiopia (8.5%) [16], and lower than estimates from Nigeria (19.6–30%) [17,18] and the sub-Saharan African average (17%) [7]. Higher mortality rates have also been reported in South Africa (23%) [19], India (47.3%) [20], and Iran (35.8%) [21], while lower rates are observed in high-income settings such as Singapore (4.6%) and Spain (4.3%) [22,23].

However, these cross-country comparisons should be interpreted with caution, as differences in healthcare infrastructure, access to specialized care, referral systems, and socioeconomic conditions substantially influence outcomes [10,11]. Mortality is shaped not only by burn severity but also by broader health system factors, including delayed presentation, financial barriers, and geographic accessibility [6,8]. These challenges are not unique to burn injuries but reflect systemic constraints in access to timely care [9,10].

The relatively lower mortality observed in this study may be explained by the younger study population and lower average burn severity (mean TBSA ≈ 18%). In contrast, studies reporting higher mortality often include older populations, larger burn sizes (TBSA ≥ 30–40%), or ICU-restricted cohorts with more severe disease [20,24,25].

### 4.1. Predictors of Mortality

Inhalation injury was identified as a strong independent predictor of mortality (AOR 6.53), consistent with previous studies [26,27,28], likely due to its association with respiratory failure, sepsis, and multi-organ dysfunction [27,29]. Based on this finding, we recommend practical strategies to reduce mortality: early recognition using clinical signs (facial burns, singed nasal hairs, carbonaceous sputum), prompt airway assessment with a low threshold for intubation, strengthening ICU capacity for ventilatory support, and training healthcare providers in early airway management at all levels.

TBSA ≥ 15% (AOR 3.33) and deep or full-thickness burns (AOR 1.97) were also significantly associated with mortality. These findings align with Ryan et al. and others [28,30]. Burns exceeding 15–20% TBSA trigger a systemic inflammatory response, immunosuppression, and increased susceptibility to infection and organ failure [31,32,33]. Deep burns destroy the skin barrier, leading to fluid loss, infection risk, and delayed healing [33,34].

ICU admission was strongly associated with mortality (AOR 14.42). However, this reflects the severity of illness rather than deficiencies in ICU care [27]. In our center, as in many others, almost all patients who die do so in the ICU because the most severely ill patients are concentrated there. In resource-limited settings, ICU admission is frequently preceded by delayed presentation and inadequate early resuscitation [27].

Referral from a health facility was associated with reduced mortality (71–82% lower odds) after adjustment for severity. Although crude mortality appeared higher among referred patients, this reflects case mix differences—more severe cases are referred. After adjustment, the protective association likely reflects early stabilization, including fluid resuscitation and wound care prior to transfer [32,34].

### 4.2. Role of Age

Age is a well-established predictor of burn mortality [29,35,36]; however, it was not significantly associated with mortality in this study, likely due to the relatively young study population (median age 18 years). This limits the generalizability of our findings to populations with a higher proportion of elderly patients [36].

### 4.3. Limitations

This study has several limitations. The retrospective design may be subject to incomplete or inaccurate documentation. The study was conducted at two referral centers and may not be generalizable to other settings. Cross-country comparisons should be interpreted cautiously due to contextual differences. Additionally, the study was not designed to compare burn mortality with other conditions, and unmeasured confounders such as pre-hospital care and exact time to presentation may have influenced outcomes.

## 5. Conclusions

This study demonstrates an in-hospital burn mortality rate of 8.5% at two major referral centers in Addis Ababa, indicating that nearly one in ten patients died after presentation. Independent predictors of mortality included ICU admission (AOR 14.42), inhalation injury (AOR 6.53), TBSA ≥ 15% (AOR 3.33), and deep dermal or full-thickness burns (AOR 1.97), while formal referral from a health facility was identified as a protective factor compared with self-referred patients. The high mortality associated with ICU admission reflects patient severity rather than deficiencies in ICU care, as critically ill patients with extensive burns and organ dysfunction are inherently at higher risk. Based on these findings, the following recommendations are proposed to guide clinical practice and public health strategies in Ethiopia: First, strengthen critical care capacity for severe burn injuries through a comprehensive assessment of ICU readiness, including staffing, equipment availability, and common causes of in-ICU mortality. Second, implement a standardized early triage and risk stratification tool incorporating TBSA ≥ 15%, inhalation injury, and deep burn depth to facilitate early identification of high-risk patients, timely resuscitation, and prioritization of critical care resources. Third, improve referral networks by creating community awareness about the importance of seeking care at nearby health centers, as timely first response, initial stabilization (including fluid resuscitation and wound care), and early referral activation can reduce mortality associated with self-referral. Finally, given the high burden of preventable burn injuries—especially among children and households relying on open-flame cooking—public health campaigns should focus on scald prevention in children and flame burn prevention in domestic settings. Addressing these priority areas has the potential to substantially reduce burn-related mortality in Ethiopia’s resource-limited setting.

## Figures and Tables

**Table 1:** Sociodemographic Characteristics of Burn Patients Admitted to AaBET and Y12MC Hospitals, Addis Ababa (1 September 2021–1 November 2023).

Variables	N or Median	% or IQR
Hospital, *n* (%)		
Y12MC	459	57.38
AaBET	341	42.62
Age, years (median, IQR)	18	23
Age groups, *n* (%)		
<10 years	288	36
10–19 years	147	18.38
20–29 years	227	28.38
30–39 years	56	7
≥40 years	82	10.26
Sex, *n* (%)		
Male	344	43
Female	456	57
Origin, *n* (%)		
Urban	694	86.75
Rural, within 2 h	20	2.50
Rural, >2 h	86	10.75
Time to care <8 h, *n* (%)	645	80.3%
First care at health center, *n* (%)	460	57.5%
ICU admission, *n* (%)	44	5.5%
Hospital stays, days (median, IQR)	21	13

**Table 2:** Burn Injury Characteristics of Patients Admitted to AaBET and Y12MC Hospitals, Addis Ababa (1 September 2021–1 November 2023).

Variables	N or Median	% or IQR
Cause of burn		
Scald	393	49.1
Flame	196	24.5
Electric	195	24.4
Contact	6	0.8
Chemicals	10	1.3
TBSA% (median, IQR)	15	14
TBSA groups, *n* (%)		
<10%	263	32.9
10–19%	311	38.9
20–29%	175	21.9
30–40%	18	2.3
>40%	33	4.1
Depth of burns, *n* (%)		
Superficial dermal	297	37.1
Deep dermal	376	47
Full thickness	127	15.9
Inhalation Injury, *n* (%)	112	14%

**Table 3:** Predictors of Mortality among Burn Patients Admitted to AaBET and Y12MC Hospitals, Addis Ababa (1 September 2021–1 November 2023).

Variable	Category	Mortality Status		COR (95% CI)	AOR (95% CI)	*p* Value
		Alive	Dead			
Degree of Burn	Superficial	467	22	1	1	
	Deep	265	46	3.69 (2.17, 6.26) *	1.97 (1.01, 3.s84) **	0.048
Inhalation	no	661	27	1	1	
	yes	71	41	14.14 (8.21, 24.36) *	6.53 (2.14–19.95) **	0.001
Mechanism of burn	Scald	378	15	1	1	
	Flame	147	49	8.40 (4.57, 15.44) *	0.88 (0.30, 2.61)	
	Electric	191	4	0.53 (0.17, 1.61)	0.404 (0.13, 1.29)	
	Chemical	6	0	0.00 (0.00, …)	0.00 (0.00, …)	
	Contact	10	0	0.00 (0.00, …)	0.00 (0.00, …)	
TBSA	<15%	373	8	1	1	
	≥15%	359	60	7.79 (3.67, 16.53) *	3.33 (1.29, 8.61) **	0.013
Place of stay in the hospital	Burn ward	444	35	1	1	
	emergency	272	4	0.19 (0.07, 0.53) *	0.41 (0.12, 1.38)	
	ICU	16	29	22.99 (11.41, 46.33) *	14.42 (5.87, 35.40) **	0.000
Head and neck involvement	Yes	360	51	3.29 (1.84, 5.88) *	1.12 (0.50, 2.54)	
	No	372	16	1	1	
Patient referral	Health center	404	56	0.41 (0.19, 0.88) *	0.29 (0.11, 0.78) **	0.014
	private clinic and primary hospital	141	8	0.00 (0.00, …)	0.00 (0.00, …)	
	Tertiary hospital other than AaBET/Yekatit	33	0	0.19 (0.07, 0.53) *	0.18 (0.053, 0.61) **	0.006
	Self-referral	154	4	1	1	

* Factors fulfill the minimum requirement during bivariate analysis. ** Factors showed statistically significant association during the multivariate analysis.

## Data Availability

The data supporting the findings of this study are available from the corresponding author upon reasonable request. The data are not publicly available due to ethical and privacy restrictions.

## Abbreviations

The following abbreviations are used in this manuscript:

AaBET	Addis Ababa Burn, Emergency, and Trauma Center
ALERT	All-Africa Leprosy, Tuberculosis and Rehabilitation Training Center
AOR	Adjusted Odds Ratio
CBHI	Community-Based Health Insurance
CI	Confidence Interval
COR	Crude Odds Ratio
HMIS	Health Management Information System
ICU	Intensive Care Unit
IRB	Institutional Review Board
IQR	Interquartile Range
LMICs	Low- and Middle-Income Countries
SPHMMC	Saint Paul’s Hospital Millennium Medical College
SIRS	Systemic Inflammatory Response Syndrome
TBSA	Total Body Surface Area
WHO	World Health Organization
Y12MC	Yekatit 12 Medical College
